# PERCEPTIONS OF ADRD CARE PARTNERS IN A VIRTUAL CO-DESIGN PROCESS

**DOI:** 10.1093/geroni/igac059.2905

**Published:** 2022-12-20

**Authors:** Nicole Werner, Anna Jolliff, Reid Parks, Anna Linden, Christian Elliott, Matthew Zuraw

**Affiliations:** Indiana University School of Public Health-Bloomington, Bloomington, Indiana, United States; Indiana University School of Public Health-Bloomington, Bloomington, Indiana, United States; University of Wisconsin - Madison, Madison, Wisconsin, United States; University of Wisconsin-Madison, Madison, Wisconsin, United States; CareVirtue, Palm Springs, California, United States; DialedIn Solutions, San Diego, California, United States

## Abstract

Participatory design, or co-design, is an effective method for creating useful digital products with older adults. However, little is known about older adults’ perceptions of virtual co-design. The purpose of this study was to explore ADRD care partner perceptions of their participation in virtual co-design, including the degree to which participants felt their participation influenced the design. After participating in a 5-session virtual co-design process, care partners completed individual semi-structured interviews and surveys focused on exploring their perceptions of the co-design process. Surveys used three sliding scale items and open text responses. Interview transcripts and survey data were analyzed using thematic analysis and descriptive statistics, respectively. Co-designers were Nf7 older adults (71% female) with a mean age of 65. On a scale of 1–100, co-designers rated the extent to which their own participation influenced the design process at a mean of 88, their fellow co-designers at 90, and the research team at 82. Thematic analysis identified five design process perceptions: 1) the process built community among co-designers; 2) sessions were well-facilitated and organized; 3) the prototype would be helpful to other care partners like them; 4) co-designers felt ownership over the prototype; and 5) co-designers had long-term goals for the product. Results suggest that care partners perceived themselves as having considerable influence on the final prototype. Those designing interventions for ADRD care partners should be encouraged that involving them in the design process may provide unanticipated benefits to care partners and build an intervention responsive to care partner needs.

